# The Effect and Mechanism of lncRNA NR2F1-As1/miR-493-5p/MAP3K2 Axis in the Progression of Gastric Cancer

**DOI:** 10.1155/2021/3881932

**Published:** 2021-07-13

**Authors:** Xiaobin Liao, Linbao Wen, Liqiong Luo

**Affiliations:** ^1^Department of General Surgery, South Hospital of Tongchuan People's Hospital, Tongchuan 727100, China; ^2^Department of Neurosurgery, The Second Affiliated Hospital of Guizhou University of Traditional Chinese Medicine, Guiyang 550003, China; ^3^Department of Oncology, Lanzhou New District First People's Hospital, Lanzhou 730300, China

## Abstract

**Background:**

LncRNA NR2F1-AS1 has been identified as an oncogene in some human tumors, such as breast cancer, nonsmall cell lung cancer, and esophageal squamous cell carcinoma. Nonetheless, whether NR2F1-AS1 is involved in the progression of gastric cancer (GC) remains unknown.

**Methods:**

The expression patterns of NR2F1-AS1, MAP3K2, and miR-493-5p in GC tissues and cells were detected by RT-qPCR. The protein expression of MAP3K2 was assessed by the Western blotting assay. The MTT assay and flow cytometry were performed to measure cell proliferation and cell apoptosis in GC cells. The transwell assay was adopted to assess cell migration in GC cells. The relationship between NR2F1-AS1, MAP3K2, and miR-493-5p was verified by a dual-luciferase reporter assay.

**Results:**

The increased NR2F1-AS1 and MAP3K2 expressions were discovered in GC tissues and cells compared with control groups. Knockdown of NR2F1-AS1 and MAP3K2 dramatically suppressed cell proliferation and migration, while it enhanced cell apoptosis in GC cells. In addition, NR2F1-AS1 was found to be a sponge of miR-493-5p, and MAP3K2 was a downstream gene of miR-493-5p. Moreover, the expression of MAP3K2 was notably reduced by miR-493-5p, and NR2F1-AS1 counteracted the inhibition of miR-493-5p.

**Conclusion:**

Thus, NR2F1-AS1 was verified to regulate GC cell progression by sponging miR-493-5p to upregulate MAP3K2 expression.

## 1. Introduction

Gastric cancer (GC) is a malignant tumor derived from the epithelium of the gastric mucosal epithelium. GC accounts for the third in all malignant tumors, the first among gastrointestinal cancer, and 95% of gastric malignancies. The morbidity and mortality of GC are the third and fifth among human malignant tumors, respectively [1]. In view of the high morbidity and mortality of GC, the study on the etiology of GC has attracted many scholars. Studies have shown that obesity, gastroesophageal reflux, unreasonable diet, *Helicobacter pylori* infection, and atrophic gastritis are all high-risk factors for GC [2–4]. At present, surgical resection of the tumor is still the main method for the treatment of GC in clinical practice [5]. But the recurrence rate of GC patients after surgery is high, leading to a poor prognosis [6]. The key of targeted therapy is to find the genes and proteins that play a key role in the development of GC and to intervene against them, so as to achieve the purpose of treatment. At present, the targeted therapy of GC has made a substantial progress [7, 8].

LncRNAs regulate gene expression at multiple levels, including changing chromatin status, binding to miRNAs and mRNAs to regulate genes' expression, and binding to RNA-binding proteins to mediate intracellular signaling [9, 10]. In recent years, great progress has been made in the research on the relationship between lncRNAs and tumor development and metastasis [11, 12]. H19 was negatively correlated with the prognosis of GC and played the carcinogenic role by regulating miRNAs and genes in GC [13–15]. PINT was found to suppress tumor progression by the crosstalk with miR-21 in GC [16]. LncRNA NR2F1-AS1 was found to play a special role in some cancers. NR2F1-AS1 was found to accelerate cell angiogenesis in breast cancer and was associated with breast cancer recurrence [17, 18]. In addition, previous research studies have verified that NR2F1-AS1 functions as an oncogene in nonsmall cell lung cancer, endometrial cancer, and esophageal squamous cell carcinoma [19–21]. On the contrary, NR2F1-AS1 suppressed cell growth by modulating miR-371-3p/TOB1 axis in colorectal cancer [22]. However, the information on the effect of NR2F1-AS1 in GC is limited.

In the current article, we first examined the expression levels of NR2F1-AS1 and MAP3K2 in GC and explored their effect on cell proliferation, cell apoptosis, and cell migration in GC. Furthermore, the regulatory network of NR2F1-AS1/miR-493-5p/MAP3K2 in GC was also explored.

## 2. Materials and Methods

### 2.1. Patients and Clinical Tissues Samples

The tissue samples of 44 GC patients at South Hospital of Tongchuan People's Hospital were collected for our experiments. Specimens from each patient included GC tissues and matched distal nontumor tissues. Before surgery, none of the patients had received any preoperative radiotherapy, chemotherapy, or immunotherapy. This study was approved by the Ethics Committee of South Hospital of Tongchuan People's Hospital. The patients or their families have signed the informed consent.

### 2.2. Cell Lines

Human normal gastric mucosa epithelial cell line (GES-1) and GC cell lines (HGC-27, AGS, MKN-74, and SNU-5) were obtained from the Typical Cell Bank of Chines Academy of Sciences (Shanghai, China). The cells were cultured in the Roswell Park Memorial Institute 1640 (RPMI-1640) medium containing 10% foetal bovine serum (FBS), penicillin, and streptomycin. The culture atmosphere of the cell incubator was 37°C, 5% CO_2_, and 95% humidity. Cell passage was carried out when the cell confluence reached 80%.

### 2.3. RT-qPCR

Total RNA was obtained from GC tissues or cells by using TRIzol. Then, the cDNA was synthesized by using a reverse transcription kit. 2 *μ*l reverse transcription product was taken for PCR reaction. The PCR reaction system was SYBR Green Master Mix 10 *μ*L, forward and reverse primers 0.8 *μ*L each, cDNA 3 *μ*L, and ddH_2_O 7.4 *μ*L. Reaction conditions were 95°C for 2 min; 95°C for 15 s, 60°C for 30 s, 72°C for 30 s, and a total of 40 cycles. GAPDH was used as an internal reference for NR2F1-AS1 and MAP3K2, while U6 was used as an internal reference for miR-493-5p. The relative expressions of NR2F1-AS1, MAP3K2, and miR-493-5p were calculated by the 2^−△△CT^ method. Primer sequences are given in Table 1.

### 2.4. Cell Transfection

AGS and MKN-74 cells were selected for the functional experiments. NR2F1-AS1 si-RNA (5ʹ-UAAUAGAAAUAUUGAGAACAU-3ʹ), MAP3K2 si-RNA, and their corresponding negative control si-NC (5ʹ-AAGACAUUGUGUGUCCGCCTT-3ʹ), pcDNA-NR2F1-AS1, and its negative control pcDNA, miR-493-5p mimic, and its negative control miR-NC were obtained from GenePharma (Shanghai, China). The transfections were carried out by Lipofectamine 3000. AGS and MKN-74 cells were cultured in an incubator at 37°C and 5% CO_2_. After transfection for 48 h, AGS and MKN-74 cells were collected for the functional experiments.

### 2.5. Western Blot Assay

The protein expression of MAP3K2 was detected by the Western blot assay. Cells in each group were collected to extract the total protein, and the protein concentration was detected by the BCA method. 30 *μ*g protein was electrophoresized by SDS-PAGE and then transferred to the PVDF membrane. Next, it was sealed with 5% skim milk for 2 h and added primary antibody (anti-MAP3K2, 1 : 1000) at 4°C overnight. After washing with TBST, secondary antibody (goat-anti-mouse, 1 : 1000) was added to incubate for 1 h. The gray values of each strip were measured by the gel imaging analysis system and Image J software.

### 2.6. Cell Proliferation

Cells were seeded in a 96-well plate with a cell density of 5 × 10^3^ cells/well. After cultured at 37°C for 1–3 days, 10 *μ*l MTT (5 g/L) was added. After incubation for 4 h, cells were added with 150 *μ*l dimethyl sulfoxide and incubated for 10 min. Cell proliferation was indirectly reflected by measuring the OD at 490 nm.

### 2.7. Cell Migration

AGS and MKN-74 cells were seeded in the upper chamber of the transwell chamber. We added 600 *μ*l Dulbecco's Modified Eagle Medium (DMEM) medium with 10% FBS to the lower chamber. The chamber was incubated in a 5% CO_2_ incubator at 37°C for 24 h. After washing with phosphate buffer saline (PBS), cells were fixed and stained by paraformaldehyde and 0.1% crystal violet. Finally, the number of migrated cells was measured by the microscope.

### 2.8. Cell Apoptosis

AGS and MKN-74 cells were centrifuged at 1000 r/min for 5 min, and the supernatant was discarded. Then, cells were added with 5 *μ*L Annexin V-FITC and PI. Finally, cell apoptosis was detected by flow cytometry after incubation at room temperature in dark.

### 2.9. Dual-Luciferase Reporter Assay

NR2F1-AS1-WT, NR2F1-AS1-MUT, MAP3K2-WT, and MAP3K2-MUT were obtained from GenePharma (Shanghai, China). Cells were transfected with miR-493-5p mimic and miR-NC, respectively. After transfection for 48 h, the luciferase activity was detected according to the requirements of the dual-luciferase reporter kit.

### 2.10. Statistical Analysis

The measurement data were presented as mean ± SD. Data were analyzed by using SPSS 21.0 statistical software. The comparison between the two groups was analyzed by the *t*-test. The chi-square test was performed to compare the two groups. *P* < 0.05 was the result of the statistical difference.

## 3. Results

### 3.1. NR2F1-AS1 and MAP3K2 Were Overexpressed in GC Tissues and Cells

**Figure 1:**
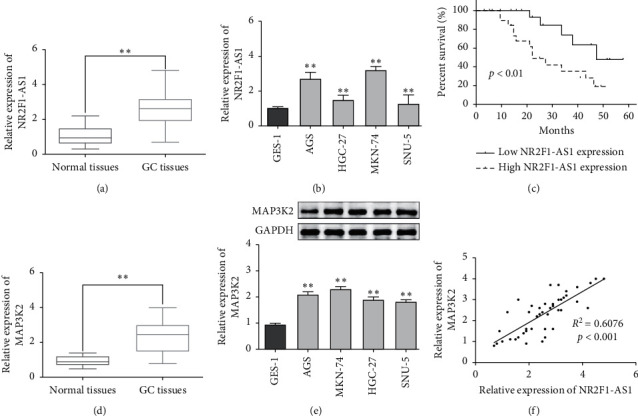
NR2F1-AS1 and MAP3K2 highly expressed in GC tissues and cells. (a, b) The expression of NR2F1-AS1 in 44 GC tissues and cell lines. (c) The relationship between NR2F1-AS1 and overall survival of GC patients. (d, e) The expression of NR2F1-AS1 in 44 GC tissues and cell lines. (f) The relationship between NR2F1-AS1 and MAP3K2 in GC tissues. ^*∗∗*^*P* < 0.01.

To investigate the role of NR2F1-AS1 and MAP3K2 in the progression of GC, the expression levels of NR2F1-AS1 and MAP3K2 were assessed by RT-qPCR. Results reflected an upward trend of NEAT1 expression in GC tissues compared with control tissues (Figure 1(a)). Next, NR2F1-AS1 was obviously upregulated in GC cells (AGS, HGC-27, MKN-74, and SNU-5) compared with GES-1 cells (Figure 1(b)). Next, we investigated the relationship between NEAT1 expression and overall survival rate. GC patients with high NR2F1-AS1 expression had a shorter survival time than those with low NR2F1-AS1 expression (Figure 1(c)). Moreover, MAP3K2 was identified as an ascending expression in GC tissues compared with control tissues (Figure 1(d)). Subsequently, MAP3K2 was highly expressed in GC cells compared to the normal cells (Figure 1(e)). Furthermore, there was a positive correlation between NR2F1-AS1 and MAP3K2 in GC tissues (Figure 1(f)). Therefore, NR2F1-AS1 and MAP3K2 may play a special role in the development of GC.

### 3.2. Knockdown of NR2F1-AS1 Repressed Cell Proliferation and Migration, While It Induced Cell Apoptosis of Cells

**Figure 2:**
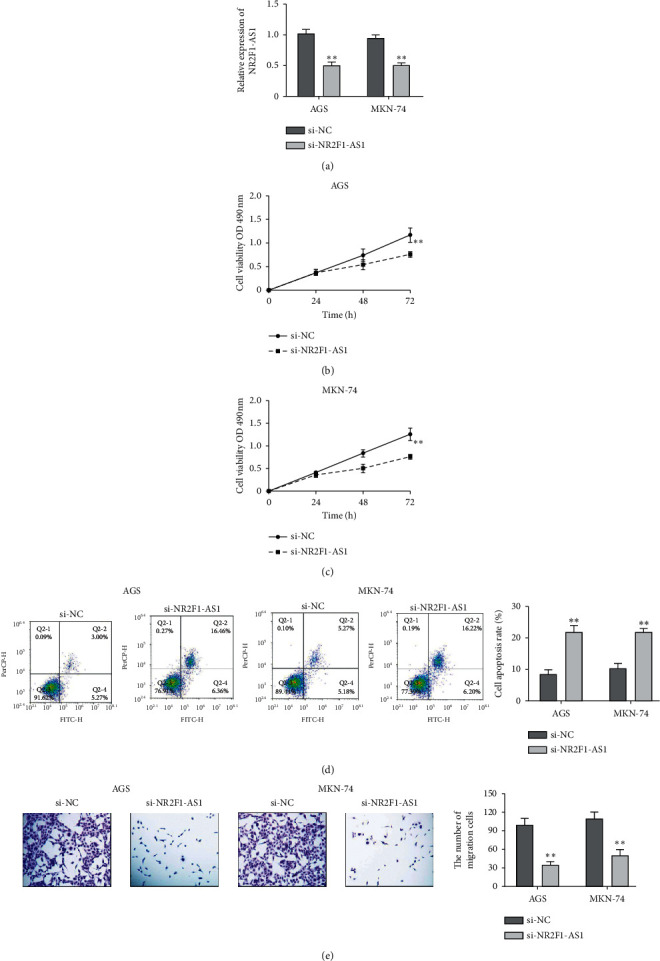
Knockdown of NR2F1-AS1 repressed cell proliferation and migration, while induced cell apoptosis of cells. (a) The expression of NR2F1-AS1 in AGS cells and MKN-74 cells transfected with NR2F1-AS1 si-RNA. (b, c) Cell proliferation in AGS cells and MKN-74 cells with si-NR2F1-AS1. (d) Cell apoptosis in AGS cells and MKN-74 cells with si-NR2F1-AS1. (e) Cell migration in AGS cells and MKN-74 cells with si-NR2F1-AS1 (scale bar = 100 *μ*m). ^*∗∗*^*P* < 0.01.

The expression of NR2F1-AS1 was significantly decreased when AGS and MKN-74 cells were transfected with NR2F1-AS1 si-RNA (Figure 2(a)). Then, the effect of NR2F1-AS1 on GC cell progression was detected by MTT, transwell assay, and FCM. As shown in Figures 2(b) and 2(c), after transfection with NR2F1-AS1 si-RNA, the OD value in GC cells decreased significantly. Compared with the control group, the migration ability of GC cells was obviously suppressed by NR2F1-AS1 knockdown (Figure 2(e)). In contrast, NR2F1-AS1 knockdown obviously accelerated the cell apoptosis rate of GC cells (Figure 2(d)).

### 3.3. Inhibition of MAP3K2 Suppressed Cell Proliferation and Migration and Enhanced Cell Apoptosis of GC Cells

**Figure 3:**
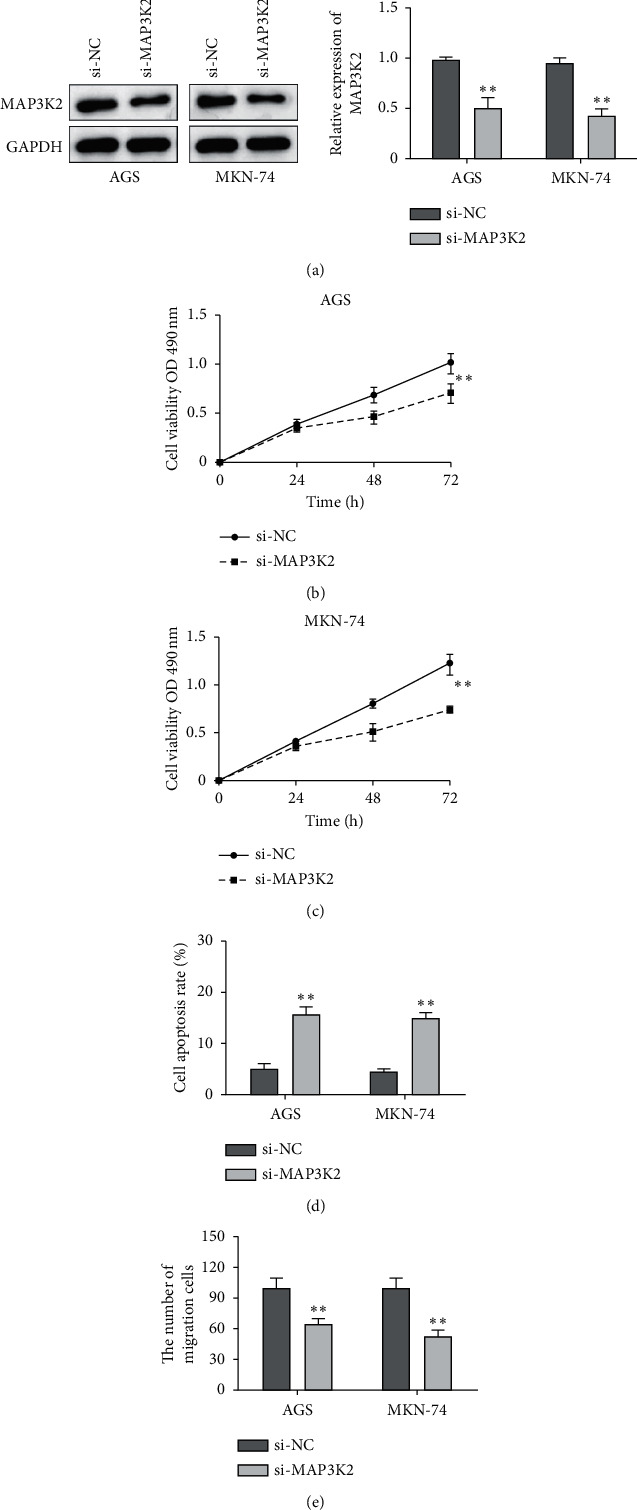
Inhibition of MAP3K2 suppressed cell proliferation and migration and enhanced cell apoptosis of GC cells. (a) The expression of MAP3K2 in AGS cells and MKN-74 cells with si-MAP3K2. (b, c) Cell proliferation in AGS cells and MKN-74 cells with si-MAP3K2. (d) Cell apoptosis in AGS cells and MKN-74 cells with si-MAP3K2. (e) Cell migration in AGS cells and MKN-74 cells with si-MAP3K2. ^*∗∗*^*P* < 0.01.

In the same way, AGS and MKN-74 cells were transfected with MAP3K2 si-RNA. As shown in Figure 3(a), the protein expression of MAP3K2 was obviously reduced after cells were transfected with MAP3K2 si-RNA. Compared with the control group, MAP3K2 knockdown notably blocked cell proliferation in GC cells (Figures 3(b) and 3(c)). Similarly, MAP3K2 knockdown suppressed GC cell migration in cells compared with the control group (Figure 3(d)). As we expected, the cell apoptosis rate was ascended when GC cells were transfected with MAP3K2 knockdown (Figure 3(e)).

### 3.4. NR2F1-AS1 Targeted miR-493-5p, and MAP3K2 Was a Target Gene of miR-493-5p in GC Cells

**Figure 4:**
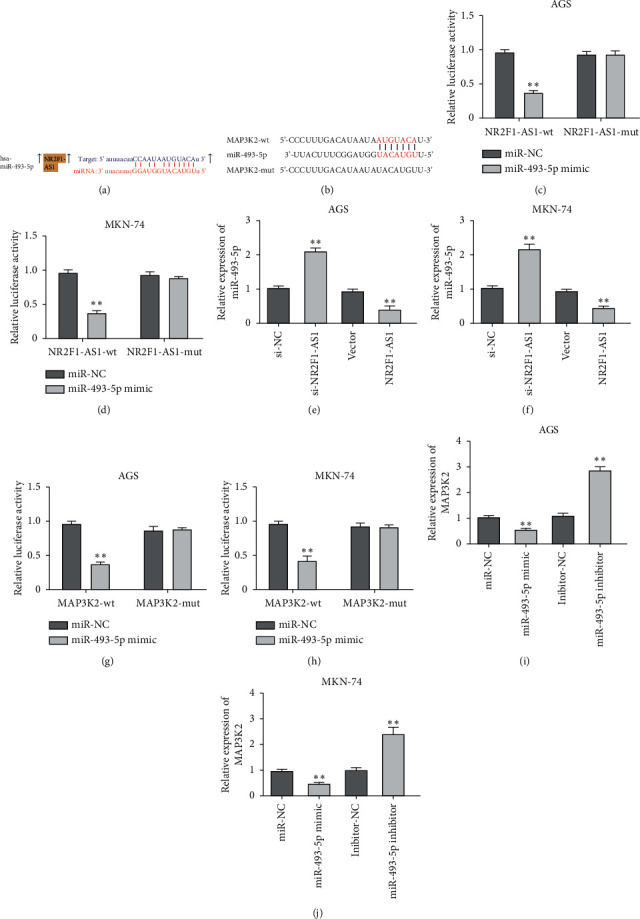
NR2F1-AS1 targeted miR-493-5p, while MAP3K2 was a target gene of miR-493-5p in GC cells. (a) The binding sites between NR2F1-AS1 and miR-493-5p. (b) The binding sites between MAP3K2 and miR-493-5p. (c, d) The effect of miR-493-5p on the luciferase activity of NR2F1-AS1-WT and NR2F1-AS1-MUT. (e, f) The expression of miR-493-5p in AGS cells and MKN-74 cells with si-NR2F1-AS1 or NR2F1-AS1. (g, h) The effect of miR-493-5p on the luciferase activity of MAP3K2-WT and MAP3K2-MUT. (i, j) The expression of MAP3K2 in AGS cells and MKN-74 cells with miR-493-5p mimic or miR-493-5p inhibitor. ^*∗∗*^*P* < 0.01.

LncRNAs have been reported to play their roles in human cancers by targeting miRNAs to regulate the downstream genes. In the current study, StarBase and TargetScan were used to predict the binding sequences between NR2F1-AS1, miR-493-5p, and MAP3K2 (Figures 4(a) and 4(b)). Then, we found that the luciferase activity of the NR2F1-AS1-WT group was notably declined after treatment with miR-493-5p mimic, while transfection of miR-493-5p mimic had no significant effect on the luciferase activity of the NR2F1-AS1-MUT group (Figures 4(c) and 4(d)). Next, RT-qPCR results displayed that the expression of miR-493-5p was increased in GC cells treated with NR2F1-AS1 knockdown, but declined when cells were treated with NR2F1-AS1 (Figures 4(e) and 4(f)). Similar experiments displayed that miR-493-5p mimic decreased the luciferase activity of MAP3K2-WT, but not MAP3K2-MUT (Figures 4(g) and 4(h)). In addition, the expression of MAP3K2 was notably descended by miR-493-5p mimic, while it was increased by the miR-493-5p inhibitor (Figures 4(i) and 4(j)).

### 3.5. Downregulation of MAP3K2 Induced by miR-493-5p Could Be Recovered by Transfection with NR2F1-AS1 in GC Cells

**Figure 5:**
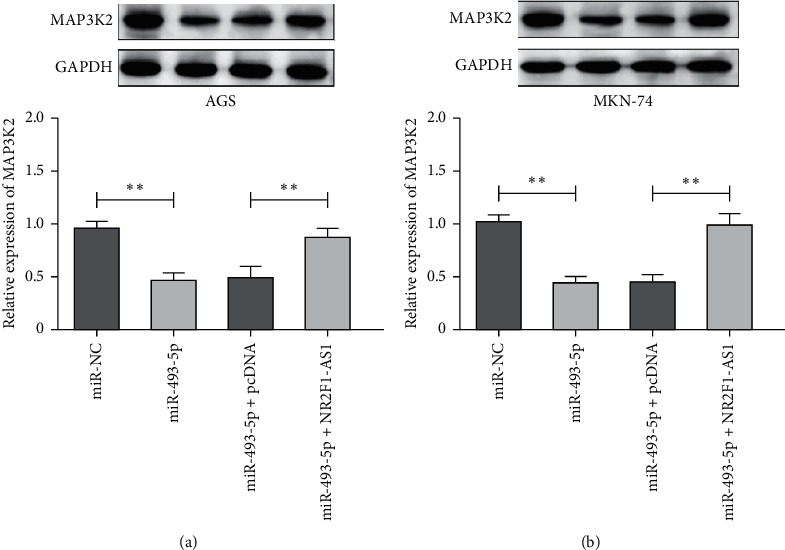
Downregulation of MAP3K2 induced by miR-493-5p could be recovered by transfection with NR2F1-AS1 in GC cells. (a) The expression of MAP3K2 in AGS cells with miR-493-5p or NR2F1-AS1. (b) The expression of MAP3K2 in MKN-74 cells with miR-493-5p or NR2F1-AS1. ^*∗∗*^*P* < 0.01.

We noticed that the protein expression of MAP3K2 was notably reduced by miR-493-5p mimic, while NR2F1-AS1 retarded the inhibiting effect of miR-493-5p on MAP3K2 expression (Figures 5(a) and 5(b)). Therefore, NR2F1-AS1 regulated GC cell progression by inhibiting miR-493-5p to upregulate MAP3K2 expression.

## 4. Discussion

NR2F1-AS1 is verified to be aberrantly expressed in several human tumors and plays an important role in the development of tumors. NR2F1-AS1 was upregulated in hepatocellular carcinoma (HCC), and downregulated NR2F1-AS1 was found to suppress cell growth and EMT progression and promote cell apoptosis of HCC cells [23]. Moreover, high NR2F1-AS1 expression was associated with poor OS, and silencing of NR2F1-AS1 blocked HCC cell hypoxia-induced glycolysis [24]. Furthermore, NR2F1-AS1 knockdown was reported to suppress cell invasion, migration, and cell growth, while it induced cell apoptosis in osteosarcoma cells by miR-483-3p and FOXA1 [25]. Whereas, the role of NR2F1-AS1 in GC is not well-understood. In our experiment, we detected the expression level of NR2F1-AS1 in GC. Moreover, functional experiments were performed to measure the effect of NR2F1-AS1 on GC progression. Additionally, the possible carcinogenic mechanism of NR2F1-AS1 in GC was discussed through molecular biology experiments.

We noticed that NR2F1-AS1 was notably overexpressed in GC tissues and cell lines. Our results were similar to previous reports. NR2F1-AS1 was highly expressed in melanoma, endometrial cancer, and cancer esophageal squamous cell carcinoma [19,  26,  27]. Furthermore, to detect the role of NR2F1-AS1 on GC progression, we knocked down the expression of NR2F1-AS1 in AGS and MKN-74 cells. Our results displayed that NR2F1-AS1 knockdown dramatically blocked cell proliferation and migration, while it enhanced cell apoptosis of GC cells. Similarly, NR2F1-AS1 downregulation was found to inhibit cell growth, migration, invasion, and the EMT process in esophageal squamous cell carcinoma cells [26]. Contrary to our findings, NR2F1-AS1 was displayed to play as an antigene in colorectal cancer and cervical squamous cell carcinoma [22, 28].

LncRNAs were reported to play vital roles in tumor progression by sponging miRNAs to regulate downstream genes. In papillary thyroid carcinoma, NR2F1-AS1 acted as an oncogene by sponging miR-423-5p to upregulate SOX12 expression [29]. In the current study, miR-493-5p was a target of NR2F1-AS1, and MAP3K was a downstream gene of miR-493-5p. Mitogen-activated protein kinase 2 (MAP3K2) is a member of the MAP3K family with known roles in activating JNK and other kinases in the MAP pathway [30, 31]. MAP3K2 is reported to be necessary for the activation of ERK1/2 and MEK1/2 [32]. We found that MAP3K2 was significantly overexpressed in GC, and silence of MAP3K2 inhibited cell proliferation and migration, but facilitated cell apoptosis in GC cells. In line with our data, MAP3K2 was reported to be an oncogene in nonsmall cell lung cancer and triple-negative breast cancer [33, 34]. Furthermore, MAP3K2 was significantly reduced by miR-493-5p mimic, while NR2F1-AS1 retarded the inhibitory effect of miR-493-5p. Finally, the network of NR2F1-AS1/miR-493-5p/MAP3K2 in GC progression was constructed.

## 5. Conclusion

In sum, NR2F1-AS1 was verified to be an oncogene in GC. Furthermore, we confirmed that NR2F1-AS1 regulated GC progression by sponging miR-493-5p to accelerate MAP3K2 expression. Therefore, our results suggested that NR2F1-AS1 might be a therapeutic target for patients with GC. However, the effect of NR2F1-AS1/miR-493-5p/MAP3K2 on the relevant pathways and the possible regulatory mechanism in the process of GC progression still need to be further studied.

## Figures and Tables

**Table 1:** Primers sequences.

Gene		Primer sequences
*NR2F1-AS1*	Forward	5′- CAGCGGTGCAAACCATGTGC-3′
	Reverse	5′- GCAAGTTGGCTGAACCAAATG-3′

*miR-493-5p*	Forward	5′- TCCTACGGAGAGGCTCAG-3′
	Reverse	5′- TCCTCGTAGTCCAACACG-3′

*MAP3K2*	Forward	5′-GCTTACGGTCTCCTGTGAGTT-3′
	Reverse	5′-AGGATTGTCTATGTCACTTCCCC-3′

*U6*	Forward	5′-CTCGCTTCGGCAGCACA-3′
	Reverse	5′-AACGCTTCACGAATTTGCGT-3′

*GAPDH*	Forward	5′-GAGTCAACGGATTTGGTCGT-3′
	Reverse	5′-TTGATTTTGGAGGGATCTCG-3′

## Data Availability

The data used to support the findings of this study are available from the corresponding author upon request.
